# Improving Soil Heavy Metal Lead Inversion Through Combined Band Selection Methods: A Case Study in Gejiu City, China

**DOI:** 10.3390/s25030684

**Published:** 2025-01-23

**Authors:** Ping He, Xianfeng Cheng, Xingping Wen, Yi Cao, Yu Chen

**Affiliations:** 1 Faculty of Land Resources Engineering, Kunming University of Science and Technology, Kunming 650093, China; heping@kmu.edu.cn (P.H.); 18437829216@163.com (Y.C.); 2 School of Fine Art and Design, Kunming University, Kunming 650214, China; 3 International Research Center of Big Data for Sustainable Development Goals, Beijing 100094, China; 4 School of Earth and Environmental Sciences, Yunnan Land and Resources Vocational College, Kunming 652501, China; chengxianfeng2020@163.com; 5 Engineering Center of Yunnan Education Department for Health Geological Survey & Evaluation, Kunming 650218, China; 6 Key Laboratory of Digital Earth Science, Aerospace Information Research Institute, Chinese Academy of Sciences, Beijing 100094, China

**Keywords:** contaminated soils, hyperspectral remote sensing, band selection methods, soil lead detection

## Abstract

Hyperspectral technology has become increasingly important in monitoring soil heavy metal pollution, yet hyperspectral data often contain substantial band redundancy, and band selection methods are typically limited to single algorithms or simple combinations. Multi-algorithm combinations for band selection remain underutilized. To address this gap, this study, conducted in Gejiu, Yunnan Province, China, proposes a multi-algorithm band selection method to enable the rapid prediction of lead (Pb) contamination levels in soil. To construct a preliminary Pb content prediction model, the initial selection of spectral bands utilized methods including CARS (Competitive Adaptive Reweighted Sampling), GA (Genetic Algorithm), MI (mutual information), SPA (Successive Projections Algorithm), and WOA (Whale Optimization Algorithm). The results indicated that WOA achieved the highest modeling accuracy. Building on this, a combined WOA-based band selection method was developed, including combinations such as WOA-CARS, WOA-GA, WOA-MI, and WOA-SPA, with multi-level band optimization further refined by MI (e.g., WOA-GA-MI, WOA-CARS-MI, WOA-SPA-MI). The results showed that the WOA-GA-MI model exhibited optimal performance, achieving an average R^2^ of 0.75, with improvements of 0.32, 0.11, and 0.02 over the full-spectrum model, the WOA-selected spectral model, and the WOA-GA model, respectively. Additionally, spectral response analysis identified 22 common bands essential for Pb content inversion. The proposed multi-level combined model not only significantly enhances prediction accuracy but also provides new insights into optimizing hyperspectral band selection, serving as a valuable scientific foundation for assessing soil heavy metal contamination.

## 1. Introduction

Mining activities can significantly impact the ecological environment, often resulting in elevated levels of heavy metals like Pb, Cd, and Hg within the soil [1,2]. These heavy metals have the potential to transfer through the food chain and be absorbed by humans, leading to possible health concerns [3,4]. Pb contamination, in particular, is a key target for monitoring due to its high toxicity and tendency to bioaccumulate. Effective management of this type of pollution requires extensive, dynamic, and large-scale monitoring [5]. Conventional methods for detecting heavy metals in soil primarily rely on laboratory chemical analyses, such as AAS and ICP-OES [6]. Although accurate, these methods demand extensive soil sampling, involve complex pre-processing, and incur significant time and expense [7]. Moreover, they are insufficient for large-scale and high-frequency soil monitoring. This has led to a growing emphasis on developing rapid, non-invasive, cost-effective, and reliable methods for the large-scale monitoring of soil heavy metal contamination [8]. Amidst this, hyperspectral remote sensing technology has become an essential approach for assessing heavy metal contamination in soils [9,10]. Hyperspectral sensing offers high spectral resolution, capturing detailed reflectance information across multiple wavelengths. These spectral data can be employed to assess and forecast heavy metal levels in the soil [11,12]. However, hyperspectral data often include hundreds to thousands of bands, many of which are redundant or weakly correlated with soil heavy metals [13,14]. Therefore, identifying relevant bands that correlate strongly with heavy metal content is essential for constructing efficient predictive models [15,16,17].

Currently, various algorithms have been developed for hyperspectral band selection and feature extraction, and these are generally classified into three main types: statistical-based algorithms, evolutionary algorithms, and heuristic optimization algorithms [18]. Statistical-based methods, like CARS, MI, and SPA, determine effective bands by analyzing inter-band correlations [19]. The CARS algorithm uses the weight information from Partial Least Squares (PLS) models to select bands, making it effective for reducing redundancy and isolating key feature bands [20]. MI calculates entropy between bands to assess correlation, emphasizing interdependencies [21], while SPA prioritizes the most representative bands to address multicollinearity [22,23]. In the domain of evolutionary algorithms, GA conducts global search optimization by simulating biological evolution, demonstrating robust search capabilities in complex spaces [12]. In heuristic optimization, WOA, which mimics whale foraging behavior, allows for rapid convergence in complex, high-dimensional spaces, efficiently locating global optima [24]. While each of these algorithms has unique advantages and has shown success in predicting soil heavy metal content, challenges remain in current research approaches. Firstly, most studies focus on single-algorithm band selection methods and their relationships with specific heavy metal elements [25,26]. Tan et al. used the CARS method to select feature bands and performed Pb content inversion, yielding R^2^ = 0.6 [9]. Chen et al. applied the TSI method for feature band selection and performed Pb content inversion, with an R^2^ value of 0.63 [15]. Single algorithms can be sensitive to noise and data complexity in band selection, which can lead to redundant band inclusion. Additionally, because optimization objectives and strategies differ across algorithms, single algorithms often fall short of meeting the dual needs of both global optimization and effective feature extraction. Secondly, although a few studies explore multiple band selection algorithms, combining two algorithms is more common. For example, Feng et al. combined WOA and SPA for feature band selection and subsequently used PLS to develop a model for Cu content inversion in soil [27]. Wei et al. combined SCA and CARS methods to establish an As content inversion model [28]. The use of three algorithms to achieve comprehensive feature extraction remains rare, particularly with a combination that integrates statistical-based, evolutionary, and heuristic algorithms to exploit their unique strengths in band selection. Finally, there has been substantial analysis of spectral response mechanisms in band extraction [29,30,31], and different studies have identified varying spectral characteristic bands for heavy metals. For instance, Zou et al. found that the characteristic bands for Pb are primarily concentrated between 450–600 nm and 870–910 nm [16], while Zhou et al. identified spectral response bands for Pb in the ranges of 570–760 nm, 920–990 nm, 1090–1260 nm, 1710–2100 nm, and 2180–2390 nm [32]. However, most of this research has centered on single-algorithm applications. There remains a notable gap in in-depth studies focused on joint band extraction across multiple algorithms.

To address the aforementioned issues, we propose a combined optimization method that integrates WOA with other band selection algorithms (CARS, GA, MI, and SPA), following the initial band selection. A multi-level feature band selection model was developed, incorporating MI for refined extraction to further optimize band combinations. Additionally, we conducted a spectral response analysis within the combined algorithm to assess the influence of specific bands on soil Pb content prediction. To evaluate the model’s effectiveness, the optimized bands were applied to predict soil Pb content in Gejiu, Yunnan, China. This study presents new perspectives and valuable references for effective band selection in hyperspectral data and for improving soil heavy metal prediction methodologies.

## 2. Materials and Methods

### 2.1. Study Area

This research was conducted in Gejiu City, situated in Yunnan Province, China. The city spans the geographic coordinates from 102°54′ E to 103°25′ E and from 23°01′ N to 23°36′ N. The region features a complex terrain characterized by low mountains and hills, along with valleys and plains. Due to its geological structure and mineralization processes, Gejiu is rich in mineral resources, particularly renowned for its tin deposits. Historically, the city has been an important mining area because of its abundant mineral wealth, evolving into a significant non-ferrous metal production base in China during the 20th century [33]. However, ongoing mining activities have introduced heavy metal contamination into the soil, with Pb levels being especially worrisome.

### 2.2. Data Collection and Content Determination

**Figure 1:**
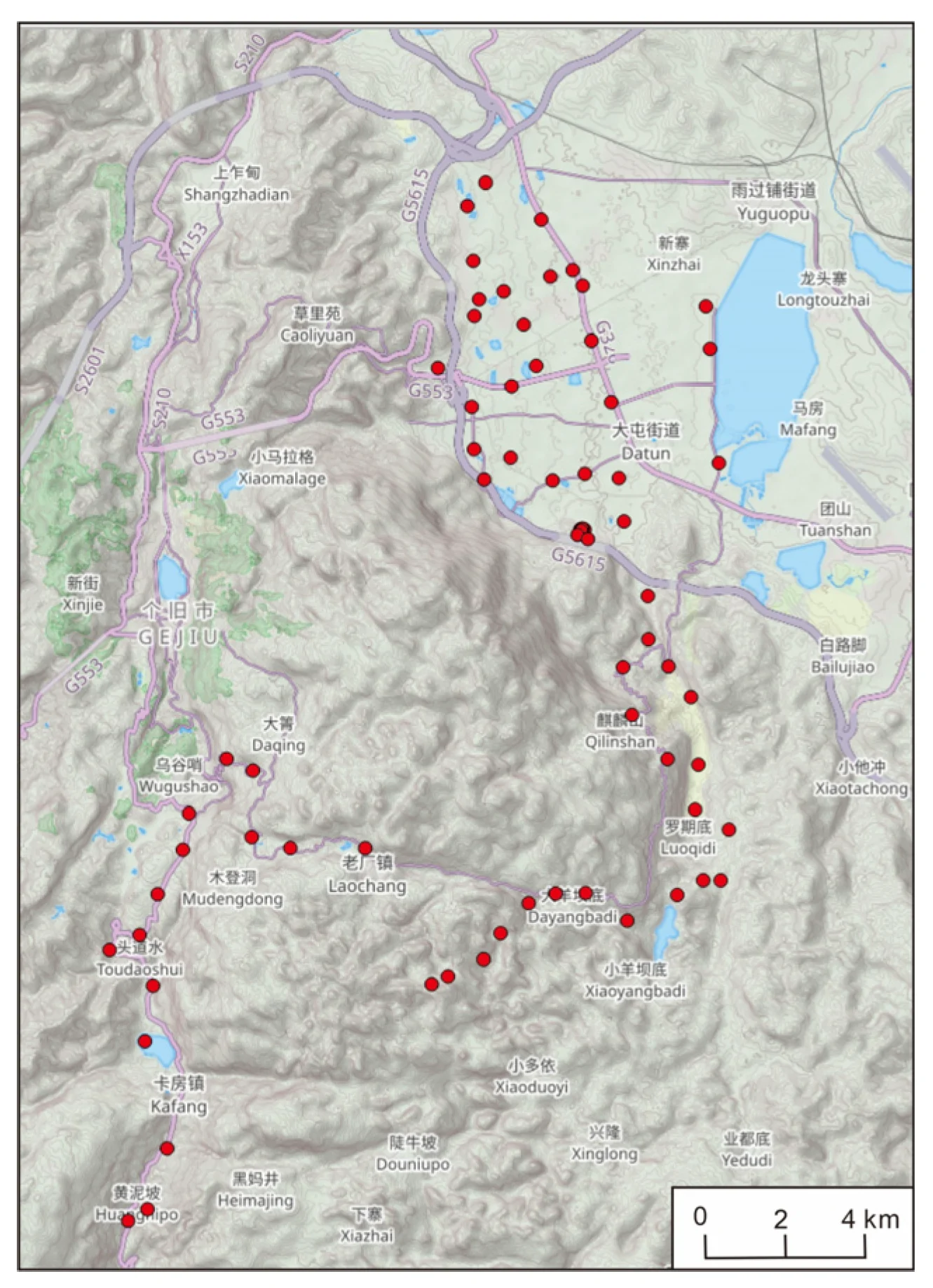
Locations of sampling points in the study area.

The soil samples were collected from 9 March to 6 April 2024, during the dry season, when the ground surface was largely exposed, creating favorable conditions for sampling. The northern part of the study area is a basin with flat terrain, where most of the land has been converted into contiguous farmland. Mining activities in this region are primarily characterized by smelting, where samples were generally collected on a 1000 m × 1000 m grid, and the sampling density increased to 500 m × 500 m around smelters. In contrast, the southern part of the study area is mountainous, with mining activities mainly involving extraction and mineral processing. Scattered farmland is found along the mining roads, where samples were collected at approximately 1000 m intervals in a linear pattern along the roads. The sampling depth was limited to 0–20 cm for the topsoil, and a total of 68 samples were collected. The distribution of sampling points is shown in Figure 1. In the laboratory, the collected soil samples were air-dried, with plant residues, gravel, and other impurities carefully removed. The samples were then ground and passed through a 100-mesh sieve (the diameter of soil particles was smaller than 0.150 mm). Each sample was split into two subsamples: one designated for Pb concentration analysis and the other reserved for spectral data acquisition. Pb levels were measured using Inductively Coupled Plasma Mass Spectrometry.

### 2.3. Spectral Measurement and Processing

Spectral data were acquired using an ASD FieldSpec 3 spectrometer, which operates within a wavelength range of 350 to 2500 nm and has a spectral resolution of 1 nm. The FieldSpec 3 is a flagship product of ASD (Analytical Spectral Devices, Inc.), now part of PANalytical B.V., headquartered in Almelo, The Netherlands. Each sample underwent five measurements, and the average of these readings was used to obtain the final spectral reflectance, ensuring the accuracy and consistency of the results. To reduce noise and minimize unnecessary interference, spectral bands from 350 to 399 nm were excluded, retaining only the 400 to 2500 nm range for further analysis.

**Figure 2:**
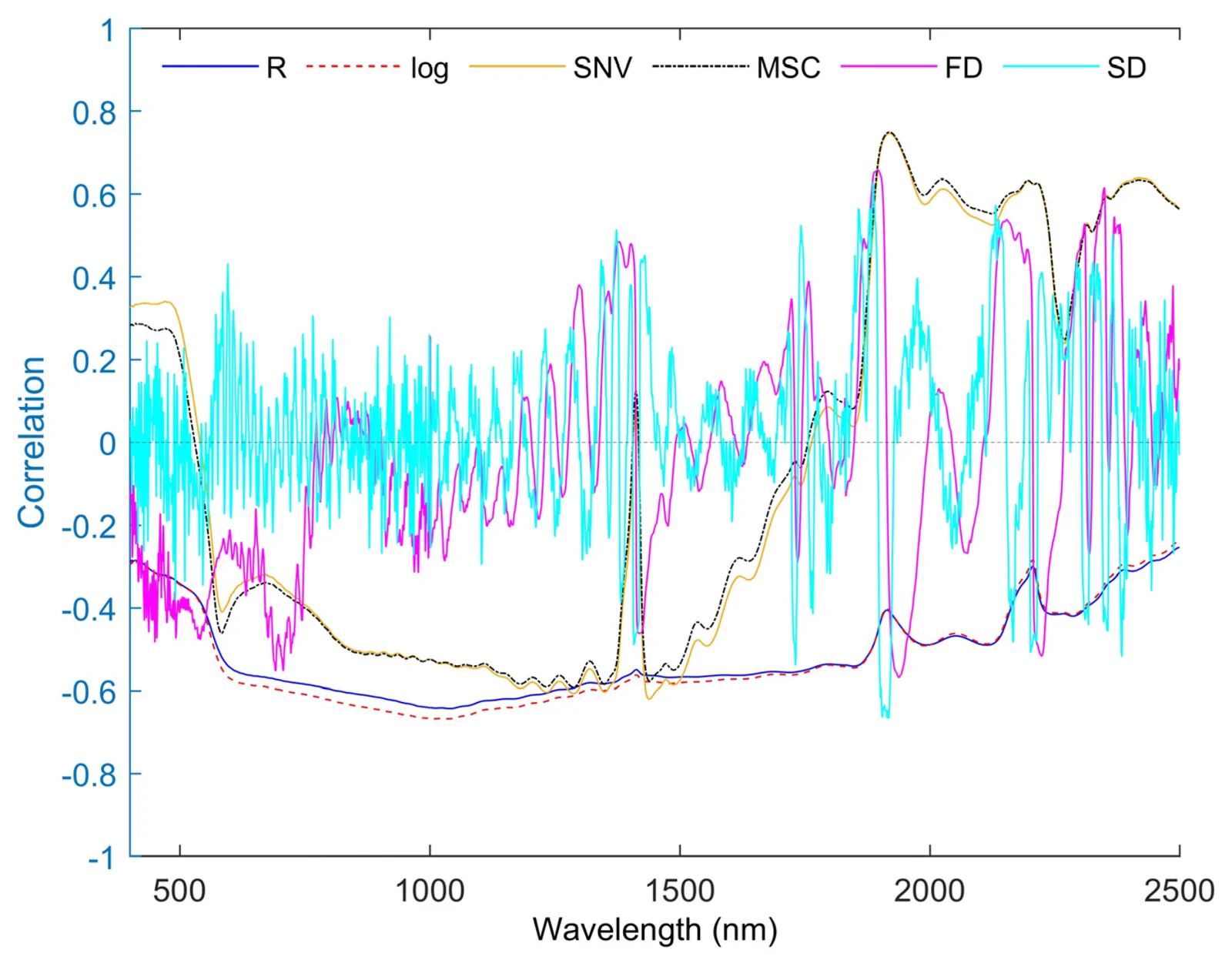
Correlation curves between different spectral preprocessing methods and soil Pb content.

Several preprocessing techniques were applied to the raw spectral data, including first derivative (FD), second derivative (SD), standard normal variate (SNV), multiplicative scatter correction (MSC), and logarithmic transformation (log). The Pearson correlation coefficient was calculated to assess which spectral transformation method showed the strongest relationship with the Pb content in the soil. Figure 2 presents the correlation between different spectral preprocessing methods and Pb content. Among these methods, the raw spectral reflectance(R) showed a strong negative relationship with Pb content over several bands, demonstrating less variability and more consistency than the other preprocessing techniques. Consequently, this study selected R as the input data for the model.

### 2.4. Band Selection and Modeling Methods

#### 2.4.1. Overview of the Research Process

The workflow of this study consists of the following steps: (1) Data Collection: Soil samples were gathered, with spectral information and Pb concentration measured for each sample. (2) Initial Band Selection and Modeling: Five methods—CARS, GA, MI, SPA, and WOA—were employed for band selection. PLS modeling was applied to each selected band to evaluate model accuracy, thereby identifying the most effective band selection method. (3) Secondary Band Combination and Modeling: Based on the preliminary results, the WOA band selection method, which demonstrated the highest accuracy, was utilized to create band combinations including WOA-CARS, WOA-GA, WOA-MI, and WOA-SPA. PLS modeling was subsequently applied to evaluate the accuracy of these combinations. (4) Final Band Combination and Modeling: Building on the secondary combinations, the MI method was further employed to extract bands, resulting in combinations such as WOA-CARS-MI, WOA-GA-MI, and WOA-SPA-MI. These combinations were subjected to PLS inversion and accuracy evaluation to obtain the optimal Pb content estimation results. (5) Common Band Extraction and Spectral Response Analysis: Common bands among the various combinations were extracted, and spectral response analysis was conducted to gain deeper insights into their inversion performance.

#### 2.4.2. Initial Band Selection

(1) CARS.

CARS simulates the “survival of the fittest” mechanism found in nature by incrementally removing bands that contribute little to the model [34]. This process effectively reduces feature redundancy and enhances model accuracy [35]. In this study, the main CARS parameters were configured with 50 sampling iterations, an alpha value of 0.95, and an epsilon of 0.01. The alpha value controls the proportion of features retained, while the epsilon value determines the convergence criterion.

(2) GA.

GA is an optimization algorithm inspired by natural selection, designed to improve feature selection through iterative refinement [36]. It optimizes band combinations using selection, crossover, and mutation operations [37]. In this study, the configuration parameters included 50 iterations, a population size of 20, and a mutation probability of 0.1.

(3) MI.

MI quantifies the relationship between random variables [38]. In the context of feature selection, MI quantifies the nonlinear correlation between each band and Pb. This study employed MI to calculate the mutual information values between each band and Pb content, allowing for the selection of bands with the highest information richness to improve the model’s predictive accuracy. An adaptive threshold mechanism was utilized to determine the optimal number of bands, thus avoiding overfitting.

(4) SPA.

SPA is a feature dimensionality reduction algorithm based on sequential forward selection [39]. It selects bands that maximize the reduction in collinearity, ensuring minimal redundancy among the selected features [40]. In this study, the SPA algorithm filtered the most representative bands from the initial set, one by one, until no further improvement was observed. This approach simplifies the data structure while retaining the bands most sensitive to variations in Pb content, thereby enhancing the model’s predictive performance.

(5) WOA.

By simulating the encircling and spiral feeding behaviors of whales, WOA conducts global optimization of feature bands [41]. For this study, WOA was configured with a maximum of 100 iterations and a population size of 30.

#### 2.4.3. Band Selection Combination

Building upon the preliminary band selection, we applied secondary optimization combinations to the initially selected bands from the WOA, aiming to maximize the advantages of each algorithm. The specific combinations are as follows:

WOA-CARS: This combination refines the feature bands identified by WOA using CARS. Due to WOA’s strong global search capabilities, it can effectively cover a wide feature space and identify potentially significant bands [42]. However, redundancy may exist among these initially selected bands. CARS addresses this by progressively eliminating less important bands and adaptively reweighting the crucial features. This combination ensures that the model simplifies the feature space while maintaining predictive accuracy and enhancing computational efficiency.

WOA-GA: This combination utilizes the genetic operations of GA (selection, crossover, and mutation) to further optimize the feature combinations based on the initial selection from WOA. GA provides good flexibility and exploratory capabilities when handling complex, high-dimensional data, allowing for a finer refinement of the feature space selected by WOA. This process identifies and retains the most explanatory bands while excluding noisy features and irrelevant information.

WOA-MI: Building on the preliminary selection by WOA, MI further extracts bands that are rich in information and highly correlated with Pb content. Incorporating MI enhances the model’s ability to capture nonlinear relationships, compensating for any nonlinear associations that WOA may not have identified during the initial screening.

WOA-SPA: This combination integrates the sequential projection characteristics of SPA to effectively reduce multicollinearity issues among the bands selected by WOA. This ensures that the final selected bands retain globally relevant information while reducing the influence of multicollinearity on the model’s performance and stability.

To further optimize the band combinations and enhance the model’s predictive capabilities, we employed MI to finely extract bands based on the aforementioned combinations, resulting in multilayer combinations such as WOA-CARS-MI, WOA-GA-MI, and WOA-SPA-MI. This three-stage combination strategy is founded on the following principles: (1) Enhancing Information Content: The band combinations after initial screening may still contain redundancy and irrelevant information; the introduction of MI can effectively extract the most explanatory bands. (2) Improving Nonlinear Capture Capability: MI can identify and retain bands with complex nonlinear relationships to Pb content, thereby improving the model’s ability to predict accurately.

#### 2.4.4. PLS Model and Evaluation Metrics

PLS is a statistical method that extracts principal components by maximizing the covariance between feature variables and target variables, thereby establishing effective predictive models [43]. In this study, the PLS method was applied to model the selected band combinations for more precise predictions of the target variable. To assess the model’s performance, we employed several evaluation metrics, including the coefficient of determination (R^2^), root mean square error (RMSE), and relative predictive deviation (RPD) [44]. R^2^ measures the extent to which the model can explain the variability in observed data, with values closer to 1 indicating a better fit [45]. Generally, an R^2^ value greater than 0.7 is considered a good model fit, while a value exceeding 0.9 is regarded as excellent. RMSE quantifies the discrepancy between predicted and actual values, where smaller values denote improved predictive accuracy. RPD quantifies the model’s ability to predict relative to the variability in the observed data, with values above 1.4 indicating good predictive performance and values greater than 2 suggesting excellent performance [46].

## 3. Results and Discussion

### 3.1. Statistical Analysis of Pb Content in Soil Samples

Table 1 presents a statistical summary of the heavy metal Pb content in the soil samples. The results show that the minimum Pb content is 34.6 mg/kg, while the maximum value reaches 9270 mg/kg, indicating significant variation in the data. The coefficient of variation (CV) of 1.65 suggests considerable data dispersion. The skewness is 2.06, indicating a right-skewed distribution, where most samples had relatively low Pb content, but a few samples exhibited much higher concentrations, resulting in outliers. The kurtosis is 3, indicating that the distribution is slightly peaked compared to a normal distribution, which suggests a certain level of concentration. The median Pb content is 428 mg/kg, further confirming the right-skewed nature of the data. These statistical parameters highlight the considerable spatial variability in the Pb content in the soil of this region. To achieve a distribution closer to normal during the inversion process, we applied a logarithmic transformation to the Pb content data.

### 3.2. Initial Selection of Characteristic Bands and Model Development

**Figure 3:**
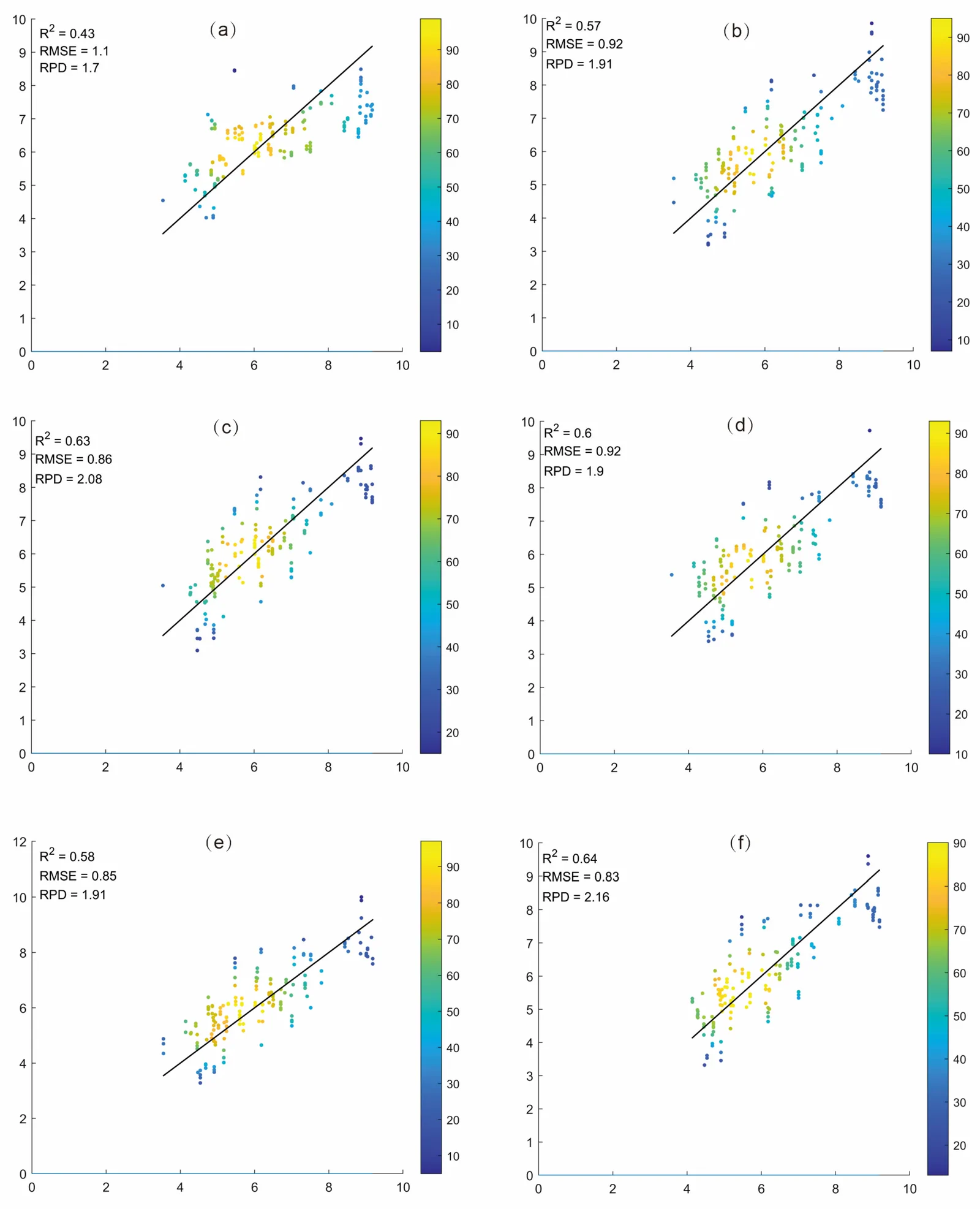
Scatter plots of the observed vs. predicted values for models based on the full spectrum and different band selection methods: (**a**) Full spectrum, (**b**) CARS, (**c**) GA, (**d**) MI, (**e**) SPA, and (**f**) WOA. Each plot’s upper left corner displays the mean test set evaluation metrics averaged over 10 runs.

We conducted initial band selection using the CARS, GA, MI, SPA, and WOA algorithms. Using both the full spectrum and these selected characteristic bands, we built prediction models with the PLS method. For model training, 75% of the 68 samples were randomly assigned to the training set, while the remaining 25% comprised the test set. To ensure result stability, the modeling process was repeated 10 times, and the arithmetic mean of the evaluation metrics from these 10 test runs was taken as the final evaluation metric. The scatter plots of model performance across the test set for each band selection method are shown in Figure 3. The results demonstrate varying degrees of accuracy improvement following feature band selection. The average R^2^ value for the full-spectrum model was 0.43, while models with bands selected by CARS, GA, MI, SPA, and WOA achieved mean R^2^ values of 0.57, 0.63, 0.6, 0.58, and 0.64, showing increases of 0.14, 0.2, 0.17, 0.15, and 0.21, respectively, compared to the full-spectrum model. In terms of RMSE, the full-spectrum model yielded a value of 1.1, while the RMSE values for each algorithm were 0.92, 0.86, 0.92, 0.85, and 0.83, showing reductions of 0.18, 0.24, 0.18, 0.25, and 0.27, respectively. For RPD, the full-spectrum model obtained a value of 1.7, while RPD values with each algorithm were 1.91, 2.08, 1.9, 1.91, and 2.16, resulting in improvements of 0.21, 0.38, 0.2, 0.21, and 0.46, respectively. In summary, WOA demonstrated superior performance in model development after band selection, achieving the highest overall accuracy among the tested methods.

### 3.3. Results of Combined Feature Bands and Model Analysis

**Figure 4:**
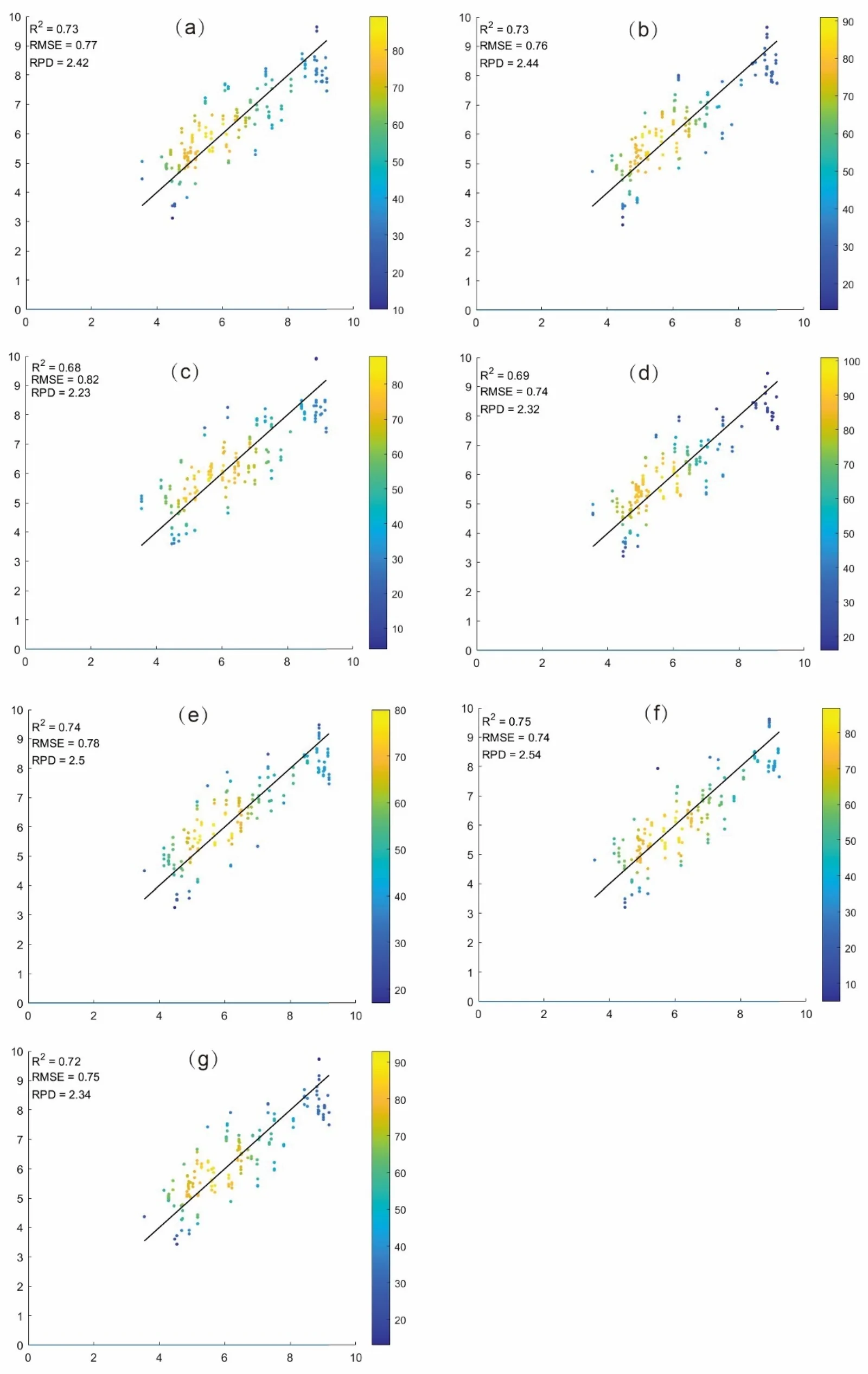
Scatter plots of real vs. predicted values for combined feature band models on the test set: (**a**) WOA-CARS, (**b**) WOA-GA, (**c**) WOA-MI, (**d**) WOA-SPA, (**e**) WOA-CARS-MI, (**f**) WOA-GA-MI, (**g**) WOA-SPA-MI. Each plot’s top-left corner shows the average test set evaluation metrics calculated from 10 runs.

Following the initial feature band selection, a second band combination was conducted using WOA, which yielded the highest accuracy, combined with other algorithms, including WOA-CARS, WOA-GA, WOA-MI, and WOA-SPA. Subsequently, PLS modeling was performed on these combined bands, following the same methodology outlined in Section 3.1. Figure 4a–d presents scatter plots of test set predictions. The results demonstrate that the combined methods—WOA-CARS, WOA-GA, WOA-MI, and WOA-SPA—improve model accuracy to varying extents compared to the standalone WOA approach. The WOA model achieved an average R^2^ of 0.64, whereas the combined methods yielded average R^2^ values of 0.73 for both WOA-CARS and WOA-GA, 0.68 for WOA-MI, and 0.69 for WOA-SPA, showing increases of 0.09, 0.09, 0.04, and 0.05, respectively. Notably, WOA-GA and WOA-CARS provided the most substantial improvements, likely because GA and CARS more effectively optimize band combinations during feature selection, reducing redundancy and better extracting information closely related to Pb content. In comparison, the WOA-MI and WOA-SPA combinations yielded smaller gains, possibly because MI does not fully utilize global information in band selection, while SPA may retain some redundant bands when selecting representative bands. For RMSE, the value for WOA alone was 0.83, while the values for the combined algorithms were 0.77, 0.76, 0.82, and 0.74, respectively, showing reductions of 0.06, 0.07, 0.01, and 0.09. This demonstrates a particular advantage of the WOA-GA and WOA-SPA combinations in reducing model error. A lower RMSE suggests greater prediction accuracy on the test set, which is crucial for real-world soil Pb content prediction and enhances the precision of contamination detection. For RPD, the WOA model had a value of 2.16, while the RPD values for the combined algorithms were 2.42, 2.44, 2.23, and 2.32, reflecting increases of 0.26, 0.28, 0.07, and 0.16, respectively. The increase in RPD indicates enhanced predictive capability, with the WOA-GA combination achieving an RPD value of 2.44, underscoring its superior ability to capture data variability.

Overall, the WOA-GA combination demonstrated the best performance in band selection for modeling. To further evaluate its effectiveness, the results of this study were compared with the existing research. Current studies on the inversion of soil heavy metal Pb content mostly employ single-band selection methods. For example, references [9,15,47] utilized different algorithms to extract characteristic bands, with their model R^2^ values around 0.60, 0.63, and 0.66, respectively. However, these studies typically rely on single evaluations, which may result in certain randomness and instability in their outcomes. In contrast, this study adopted a multi-band combination optimization method, incorporating 10-fold iterative calculations to enhance the robustness of model evaluation. After optimization using the WOA-GA combination, the model’s average R^2^ value increased to 0.73, outperforming single-band selection methods. This indicates that the multi-band combination algorithm can extract key bands more effectively related to Pb content, while significantly reducing band redundancy, thereby improving both the predictive accuracy and reliability of the model.

Although similar multi-band optimization algorithms are rarely applied in Pb content prediction studies, relevant attempts have been made in the inversion of other soil components. For instance, reference [27] combined WOA and SPA methods to predict Cu content, achieving a model R^2^ value of approximately 0.8. Reference [21] used a combination of ACO and MI methods to predict soil total TN content, achieving an R^2^ value of 0.96. These studies demonstrate that multi-band optimization algorithms have broad applicability and potential in soil component inversion. The successful application of the WOA-GA combination algorithm in this study further validates its advantages and potential for wider use in Pb content prediction.

### 3.4. Final Combined Feature Band Results and Analysis

**Figure 5:**
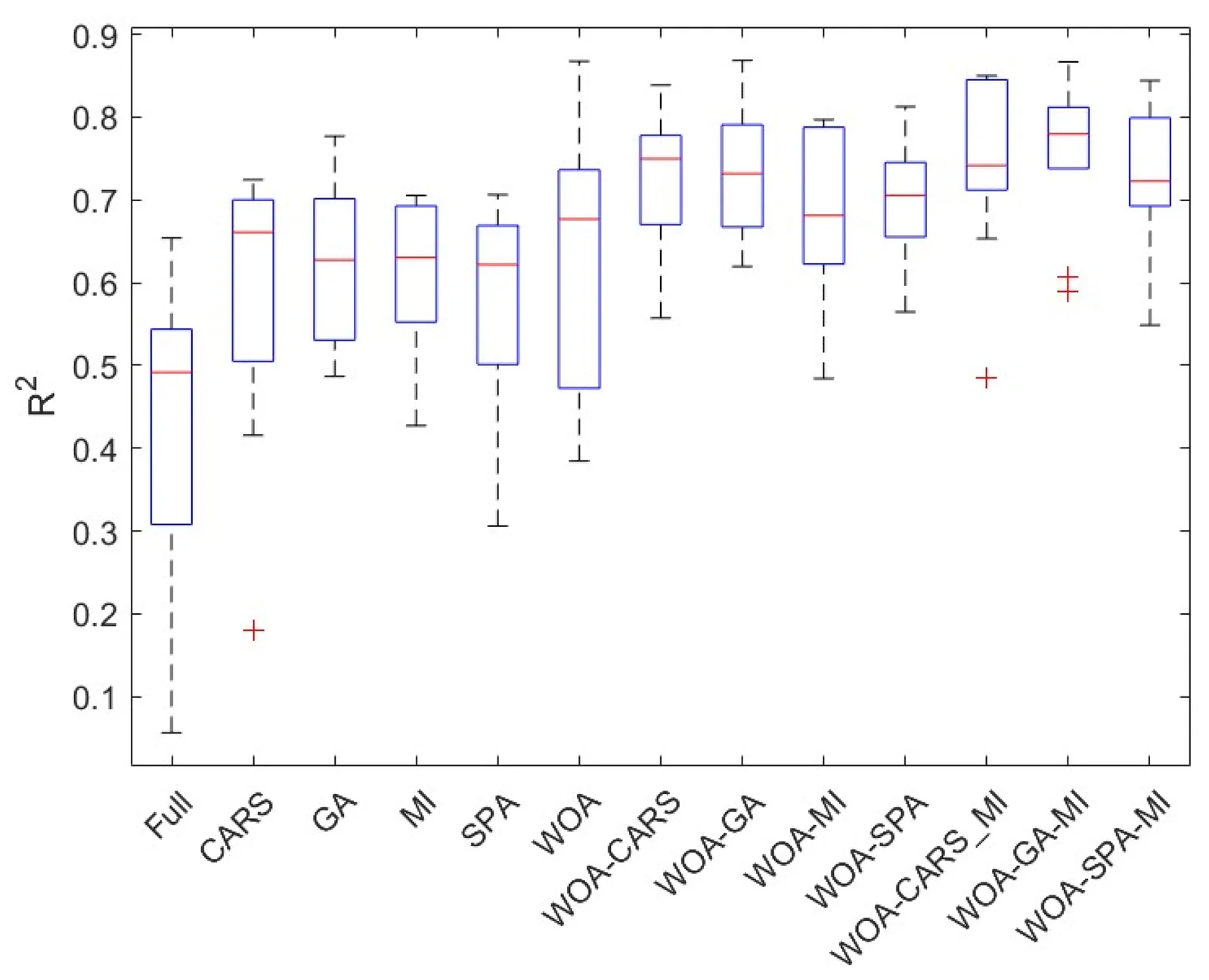
Comparison of R^2^ values between full-band modeling and band combination modeling (with “+” indicating outliers).

To further investigate the effectiveness of band combinations in enhancing model predictive ability, we applied MI for refined band selection based on previous combinations, forming multi-level combinations: WOA-CARS-MI, WOA-GA-MI, and WOA-SPA-MI. The combined bands were modeled using PLS, with their test set performance displayed in Figure 4e,f. Figure 5 presents a comparison of R^2^ values for all band combinations and the full-band model.

**Figure 6:**
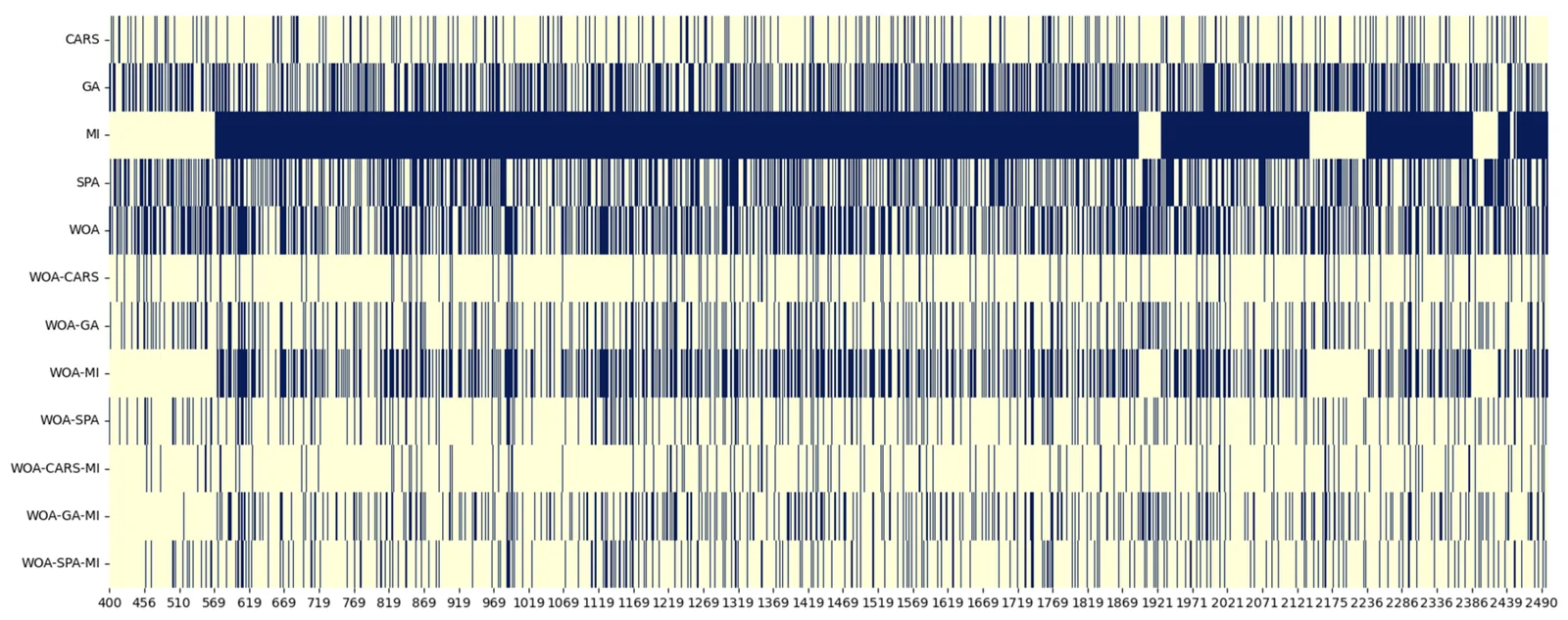
Comparison of characteristic bands extracted by different band selection methods.

The results show that the average R^2^, RMSE, and RPD values for WOA-CARS-MI were 0.74, 0.78, and 2.50, respectively, indicating a slight improvement over WOA-CARS’s 0.73, 0.77, and 2.42. However, outliers can be observed in the box plot in Figure 6, which may indicate that the model’s predictive ability is affected in certain cases.

For WOA-GA-MI, the average R^2^, RMSE, and RPD were 0.75, 0.74, and 2.54, respectively, showing improvements compared to WOA-GA’s values of 0.73, 0.76, and 2.44. However, the presence of outliers in WOA-GA-MI suggests performance variability in certain cases.

In the case of WOA-SPA-MI, the average R^2^, RMSE, and RPD were 0.72, 0.75, and 2.34, which represents a limited improvement compared to WOA-SPA’s values of 0.69, 0.74, and 2.32. This indicates that the WOA-SPA-MI feature selection approach did not significantly enhance predictive performance in this optimization.

Overall, while the MI application improved certain combinations, the presence of outliers indicates the need to consider potential data anomalies when evaluating model performance. Additionally, although WOA-GA-MI showed limited improvement in predictive performance, its optimization approach provides valuable insights for future research.

Based on these results, WOA-GA-MI demonstrated good predictive performance in the sampling area of this study. However, its applicability to areas beyond the study region requires further validation. The distribution of soil heavy metal Pb is influenced by multiple factors, such as geological background, mining activities, and soil types, which may vary significantly across different regions. Therefore, future research could consider expanding the sampling range to include more diverse environmental conditions, further evaluating the generalization performance of the multi-level optimization method and its applicability in more complex soil environments.

### 3.5. Spectral Response Mechanisms of Sensitive Bands Selected by Different Methods

Different band selection techniques resulted in varying numbers of selected bands: CARS, GA, MI, SPA, WOA, WOA-CARS, WOA-GA, WOA-MI, WOA-SPA, WOA-CARS-MI, WOA-GA-MI, and WOA-SPA-MI selected 304, 1027, 1752, 965, 1018, 173, 502, 850, 306, 166, 469, and 296 bands, respectively (see Figure 6). Significant variations were observed in the wavelength ranges chosen by these methods. For example, the WOA and GA methods tend to prioritize specific bands with strong responses within the spectral region, whereas CARS and MI focus more on characteristic bands that are significantly associated with Pb content. These differences may stem from the unique optimization goals and characteristics of each method.

Next, we identified 22 common bands selected by the WOA-CARS-MI, WOA-GA-MI, and WOA-SPA-MI combinations, including the wavelengths 590, 866, 996, 1115, 1144, 1169, 1494, 1555, 1719, 1770, 1909, 1977, 1992, 2011, 2132, 2165, 2166, 2187, 2353, 2392, 2453, and 2486 nm. The spectral response characteristics of these bands suggest the following associations with soil constituents influencing Pb distribution: (1) 590 nm and 996 nm are likely linked to oxides, such as iron oxides [32], which affect Pb mobility and distribution, making these bands significant for Pb inversion; (2) 866 nm and 1115 nm correlate with soil moisture and organic matter response [48], where Pb content may vary with changes in these factors; (3) 1144 nm and 1494 nm are associated with soil organic matter, which can form complexes with Pb, affecting the spectral signal; (4) 1555 nm relates to organic matter and clay minerals in soil [49], components that can impact Pb distribution; (5) 1719 nm, 1770 nm, and 1977 nm are linked to hydrated minerals, such as clay minerals containing OH groups [50], which play a role in Pb adsorption; and (6) 2165 nm, 2187 nm, 2353 nm, and 2486 nm reflect clay and carbonate mineral responses [50].

## 4. Conclusions

(1) In this study, we applied CARS, GA, MI, SPA, and WOA to the original spectrum for initial feature band selection and constructed prediction models for Pb content in topsoil using the PLS algorithm. The results indicate that models using selected feature bands outperformed the full-spectrum model, with the WOA-based Pb prediction model achieving the highest performance, yielding an average R^2^ of 0.64 on the test set, representing an improvement of 0.21 over the full-spectrum model.

(2) To further leverage each algorithm’s strengths based on the initially selected bands, we developed a combined feature band selection method based on WOA with CARS, GA, MI, and SPA (WOA-CARS, WOA-GA, WOA-MI, WOA-SPA). Models built with these combined band selection methods outperformed those using single algorithms, with the WOA-GA combination achieving the highest average R^2^ of 0.73, representing an improvement of 0.09 over WOA alone and 0.3 over the full-spectrum model. This highlights the considerable benefit of using combined algorithms to improve model accuracy.

(3) To further refine band selection and enhance prediction accuracy, this study applied the mutual information (MI) algorithm to the above combinations, forming multi-level combined models, including WOA-CARS-MI, WOA-GA-MI, and WOA-SPA-MI. The results show that incorporating MI improved performance in some combined models. However, outliers were observed in the WOA-CARS-MI and WOA-GA-MI models, indicating the need for careful outlier treatment in model evaluation. Notably, the proposed WOA-GA-MI model showed the most significant improvement, achieving an average R^2^ of 0.75, which is an increase of 0.32, 0.11, and 0.02 compared to the full-spectrum, WOA feature spectrum, and WOA-GA models, respectively.

(4) Based on the band selection results of the above multi-level combined models, 22 common bands were extracted from the WOA-CARS-MI, WOA-GA-MI, and WOA-SPA-MI models, namely: 590, 866, 996, 1115, 1144, 1169, 1494, 1555, 1719, 1770, 1909, 1977, 1992, 2011, 2132, 2165, 2166, 2187, 2353, 2392, 2453, and 2486 nm. Spectral analysis reveals that different bands correspond to distinct spectral features of various soil components. Some wavelengths are linked to the reflectance of soil oxides, such as iron oxides at 590 nm and 996 nm. Others are associated with soil organic matter content, with bands at 1144, 1494, and 1555 nm. Additionally, certain wavelengths are related to the presence of clay minerals or hydroxyl (OH) groups (e.g., 1719, 1770, and 1977 nm), as well as clay and carbonate minerals (e.g., 2353 nm and 2486 nm). These findings provide a basis for a deeper understanding of soil spectral characteristics.

## Figures and Tables

**Table 1:** Statistical summary of measured heavy metal Pb content.

Sample Size	Min *	Max *	Mean *	SD *	CV	Skew	Kurt	Median *
68	34.6	9270	1555.93	2567.11	1.649885	2.06	3	428

* mg Pb/kg soil.

## Data Availability

The data presented in this study are available on request from the corresponding author.
